# Acceptable Nomenclature for Pregnancy Loss Care: A Cross‐Sectional Observational Survey

**DOI:** 10.1111/1471-0528.70057

**Published:** 2025-10-11

**Authors:** Beth Malory, Louise Nuttall, Alexander E. P. Heazell

**Affiliations:** ^1^ Survey of English Usage University College London London UK; ^2^ School of English University of Nottingham Nottingham UK; ^3^ Maternal and Fetal Health Research Centre, Division of Developmental Biology and Medicine, Faculty of Biology, Medicine and Health University of Manchester Manchester UK; ^4^ Department of Obstetrics, Saint Mary's Hospital Manchester University NHS Foundation Trust Manchester UK

**Keywords:** baby loss, ectopic pregnancy, miscarriage, nomenclature, pregnancy loss, stillbirth, terminology

## Abstract

**Objective:**

To conduct a pilot study evaluating the acceptability of pregnancy loss nomenclature among people with recent lived experience and make recommendations for UK mass communication.

**Design:**

Electronic internet‐based questionnaire.

**Setting:**

UK.

**Population or Sample:**

Service users who accessed UK healthcare for > 1 experience(s) of pregnancy loss between 2021 and 2024 (*n* = 391).

**Methods:**

Descriptive and inferential statistics.

**Main Outcome Measures:**

Acceptability ratings for pregnancy loss nomenclature used diagnostically in UK healthcare settings.

**Results:**

Much nomenclature currently in use in UK pregnancy loss care was rated ‘unacceptable’ by a majority of study participants. *Spontaneous abortion, incompetent cervix*, *and cervical incompetence* were among the terminology rated as ‘unacceptable’ by > 80.0% of the respondents rating terms for the process of loss. In contrast, *pregnancy loss* and *ectopic pregnancy* were rated ‘acceptable’ by > 80.0% of respondents. As nomenclature for pregnancy loss outcomes, *products, contents of the womb/uterus*, and *tissue* were rated ‘unacceptable’ by > 80.0% of respondents. *Baby* and ‘their given name’ were rated ‘acceptable’ by > 80.0% of respondents across all gestational age brackets. Some terminology elicited mixed acceptability ratings.

**Conclusions:**

Some pregnancy loss nomenclature attracted consensus acceptability or unacceptability ratings for respondents. The data inform evidence‐based recommended alternatives, which should be adopted for mass communications relating to pregnancy loss.

## 1 Introduction

Recent research has begun to demonstrate empirically the detrimental impacts language can have on perceptions and experiences of reproductive healthcare [1, 2, 3]. This echoes clinicians' calls, both recent [4, 5, 6] and historical [7, 8], for terminology reform in maternity contexts. Such calls highlight pregnancy loss terminology, including *spontaneous abortion, incompetent cervix* and *miscarriage*, as harmful, as do consensus statements produced following consultation between clinicians [9, 10, 11]. These summarise perceived issues with diagnostic nomenclature and recommend alternatives. In recent years, language guidelines such as the Royal College of Obstetricians and Gynaecologists' (2022) have also sought ‘to create consistency, fairness and inclusivity’ via recommended terminological substitutions [12].

However, the scarcity of empirical data has made such recommendations inconsistent, often contradictory, and lacking an evidence base; for example, the use of *anembryonic pregnancy* is both encouraged [11] and discouraged [9]. Such inconsistencies highlight the need for language guidance which reflects not only clinical expertise but also empirical data on language usage in contemporary healthcare settings and variation in service users' preferences.

The Supporting Policymakers to Negotiate Communication Challenges around Pregnancy Loss (SuPPL) project used an empirical, quantitative pilot study to identify pregnancy loss terminology used in UK health settings that is clearly acceptable or unacceptable to service users. This approach distinguishes SuPPL from the core activities of its parent project, Engaging Stakeholders to Explore Linguistic Challenges in Communicating about Pregnancy Loss (EStELC) [3], which gathered qualitative data on experiences of language during pregnancy loss. EStELC findings highlighted significant variation among participants with lived experience of pregnancy loss in terminology considered helpful or unhelpful.

The EStELC project's chief recommendation was that individual language needs of people experiencing pregnancy loss be respected and accommodated in clinical settings wherever possible. However, this leaves uncertainty as to nomenclature for what might be called ‘mass communication’ settings, such as policy, websites and leaflets used for public health messaging, which are patient‐facing but where respecting individual language needs is clearly unfeasible. The SuPPL project therefore aimed to establish a standardised set of words that would minimise harm and confusion for the majority in these contexts [13].

## 2 Methods

### 2.1 Patient and Public Involvement

People with lived experience of pregnancy loss contributed to the development of the EStELC project and its subsidiary SuPPL. Recruited via social media, with assistance from partner charity organisations, they contributed via written contributions and focus groups. Clinicians, clinical academics and charity collaborators formed an Expert Advisory Group (see Acknowledgements), which met at symposia facilitating conversations about challenging aspects of pregnancy loss nomenclature in 2023 and 2024. These contributions led to SuPPL as a sub‐project focused on identifying least problematic terminology for mass communication contexts.

### 2.2 Survey Design and Dissemination

Data are from an online survey conducted between April and July 2024. The survey comprised 20 questions, each asking if the respondent had experienced a specific type of loss during the study period. For the type(s) of experience to which they answered ‘yes’, each respondent was asked structured sub‐questions about their experience and the language used. Respondents were asked about their recollection of terminology used to describe (a) their loss experience and (b) their baby, within healthcare settings and, separately, in other contexts. Participants rated the acceptability of terminology on a 7‐point Likert scale, where 1 was ‘Totally unacceptable’ and 7, ‘Perfectly acceptable’. The survey also included a section on participants' characteristics and the number and type of pregnancy loss experiences but collected no identifying details.

The questionnaire link was distributed online via social media, with the support of national charities and grassroots organisations across the UK. Participants consented to take part voluntarily by reading the information sheet and completing a consent form at the start of the survey. A power calculation was used to determine the sample size of > 384 for the pilot study. To estimate parameters for the power calculation, we used the estimated number of pregnancy losses occurring in the UK in a given calendar year [14], with a sampling error margin at ±5% at the 95% confidence level.

### 2.3 Survey Participants

Individuals who had experienced pregnancy loss of any kind (spontaneous loss at any gestation, medically or surgically managed losses, ectopic pregnancy, molar pregnancy, Termination for Medical Reasons) between April 2021 and July 2024 were eligible to participate, providing they had accessed healthcare in the UK because of their loss. This period for eligibility was selected to optimise the accuracy of recall and to ensure current nomenclature usage would be reflected.

Participants were invited to answer only those questions relating to terminology used (1) around loss during the gestational ‘bracket(s)’ of their own loss(es) in the specified period, or (2) that were specific to a particular type of loss (e.g., ectopic or anembryonic pregnancy). The gestational brackets used to divide questions were < 5^+6^, 6^+0^–9^+0^, 9^+1^–13^+6^, 14^+0^–17^+6^, 18^+0^–23^+6^, 24^+0^–29^+6^, 30^+0^–39^+6^ and 40^+0^ weeks onwards. Where participants had experienced multiple losses at different stages of pregnancy within the specified period, they were invited to consider each experience and its terminology separately.

### 2.4 Analysis

Although the frequency of recall is beyond the scope of this paper, for acceptability analysis we considered only nomenclature recalled by at least 10.0% of participants in relation to a given gestational bracket or type of loss.

Acceptability rating responses were divided into three categories: ‘Unacceptable’ (1–3 on the Likert scale outlined above), ‘Neutral’ (4) and ‘Acceptable’ (5–7). The frequency of ratings in each category (see Tables S1 and S2) was compared using descriptive statistics. Mean percentages were compared for the acceptability of different nomenclature and for acceptability of its use in relation to losses occurring across different gestational periods. In the inferential analysis, Kolmogorov–Smirnov and Shapiro‐Wilk tests determined that the data were not normally distributed, and paired rating scores (1–7) were therefore subjected to a Wilcoxon signed‐rank test. A *p* value threshold of < 0.05 was taken to signify a statistically significant difference in two‐tailed tests. Analysis was conducted using SPSS Statistics 29 for Mac.

## 3 Results

### 3.1 Study Population

The survey was initiated by 664 respondents and was completed in full by 391 people. More than half (54.7%, *n* = 208) were aged between 25 and 34 years, and most (96.8%, 368) were female (Table 1). Percentages in Table 1 have been weighted to reflect sample sizes for each gestational age group. This compensates for limitations in available data on the incidence of pregnancy loss, particularly prior to 24^+0^ weeks' gestation [15, 16], as well as limitations associated with the use of volunteer sampling, which may mean that the distribution of responses across gestational age groups in the survey population may not align with the actual distribution in the wider population.

**TABLE 1:** Background characteristics reported by survey respondents.

Characteristic	Survey respondents; % (total no. respondents to question)
Age (years)	
18–24	3.4 (13)
25–34	54.7 (208)
35–44	39.7 (151)
45–54	2.1 (8)
55–64	0 (0)
65–74	0 (0)
Gender identity	
Woman	96.6 (365)
Man	3.2 (12)
Non‐binary	0.5 (2)
Genderqueer	0 (0)
Questioning or unsure	0 (0)
Sex	
Female	96.8 (368)
Male	3.2 (12)
Intersex	0 (0)
First language	
English	91.1 (346)
Other	7.6 (29)
Left blank	1.3 (5)
Total number of experiences of pregnancy loss (including pre‐April 2021)	
1	46.8 (178)
2	27.6 (105)
3	9.7 (37)
4+	12.6 (48)
Left blank	1.1 (4)
Experienced loss of one or more pregnancies since April 2021 at	
Pre 5 weeks	34.4 (130)
6–9 weeks	37.2 (139)
9–13 weeks	20.3 (76)
14–17 weeks	9.6 (36)
18–23 weeks	14.1 (53)
24–29 weeks	5.1 (19)
30–39 weeks	7.8 (29)
40+ weeks	3.2 (12)
Experienced loss of one or more pregnancies where	
Ultrasound showed a pregnancy sac but no baby was visible	23.3 (88)
The baby had implanted outside the uterus	15.1 (57)

### 3.2 Unacceptable Terminology

We categorised acceptability and unacceptability of terms according to the degree of consensus among respondents (see Tables S1 and S2). Words and phrases with a clear consensus on unacceptability (rated ‘unacceptable’ by 80.0%–100.0% of respondents) included *spontaneous abortion, incompetent cervix* and *products of conception* (Table 2).

**TABLE 2:** Words and phrases rated ‘unacceptable’ in the SuPPL dataset ordered by degree of consensus.

Mean % rating	Unacceptable words/phrases (*n* = total number of responses)			
80%–100% (clear consensus)	For the experience		For the baby	
	Spontaneous abortion	88.9%, *n* = 269	Products	95.6%, *n* = 412
	Incompetent cervix/cervical incompetence	83.2%, *n* = 118	Mass	91.5%, *n* = 328
	Cervical insufficiency	81.1%, *n* = 116	Tissue	88%, *n* = 416
			Products of conception	87.1%, *n* = 419
			Contents of the womb/uterus	82.4%, *n* = 415
			Cells	82.0%, *n* = 331
65%–80% (apparent consensus)	Intrapartum fetal death	70.1%, *n* = 127	Pregnancy tissue	74.5%, *n* = 420
	Intrauterine death	66.1%, *n* = 127	Fetus (from 14 weeks' gestation)	73.7%, *n* = 137
			Non‐viable (baby)	72.1%, *n* = 412
50%–65% (marginal consensus)	Intrapartum stillbirth	62.7%, *n* = 126		
	Miscarriage (when used for loss 18–23 weeks' gestation)	61.2%, *n* = 49		
	Empty sac	60.2%, *n* = 190		
	Biochemical/chemical pregnancy	59.9%, *n* = 125		
	Blighted ovum	58.3%, *n* = 185		
	Fetal anomaly/fetal anomalies	58.0%, *n* = 131		
	Non‐viable (pregnancy)	57.7%, *n* = 402		
	Fetal death	52.6%, *n* = 135		
	Fetal loss	50.1%, *n* = 135		

*Spontaneous abortion* was rated ‘unacceptable’ by 88.9% of respondents who had experienced loss between 6^+0^ and 23^+6^ weeks of pregnancy. *Incompetent cervix* and *cervical incompetence* were both considered unacceptable by 83.3% and 83.1% of respondents who experienced loss at 14^+0^–17^+6^, 18^+0^–23^+6^, 24^+0^–29^+6^ and/or 30^+0^–39^+6^ weeks, respectively. Acceptability of *cervical insufficiency* was not markedly different from that for *incompetence* at these gestations, with a mean unacceptability rating of 81.1%. The Wilcoxon signed‐rank test showed no significant difference in acceptability of this term compared to *cervical incompetence* (*p* = 0.19) or *incompetent cervix* (*p* = 0.21).

For descriptions of what participants perceived they had lost, words and phrases with a clear consensus on unacceptability (rated ‘unacceptable’ by 80.0%–100.0% of respondents) included *products* (95.6%), *tissue* (88.0%), *products of conception* (87.1%) *and contents of the womb/uterus* (82.4%) for losses in each gestational bracket up to 23^+6^. *Cells* and *mass*, often used in the context of Pregnancy of Unknown Location [17, 18], were rated ‘unacceptable’ by 82.0% and 91.5% of respondents who experienced a loss up to 13^+9^ weeks, respectively.

Words and phrases rated ‘unacceptable’ by 65.0%–79.0% of respondents included *intrapartum fetal death, intrauterine death* and *pregnancy tissue* (Table 2). *Intrapartum fetal death* and *intrauterine death* were rated ‘unacceptable’ in the gestational brackets between 14^+0^–40^+0^ weeks' gestation with means of 70.1% and 66.1%, respectively. *Pregnancy tissue* was rated ‘unacceptable’ by 74.5% of respondents. When used for losses from 14^+0^ weeks, an increasing majority rated *fetus* ‘unacceptable’ with each gestational bracket. On average, 73.7% of respondents with experience of loss after 14^+0^ weeks rated *fetus* ‘unacceptable’, with 94.3% rating it ‘unacceptable’ after 24^+0^ weeks.

Words and phrases deemed ‘unacceptable’ by only a marginal majority (50.0%–64.0% of respondents) included *intrapartum stillbirth, empty sac* and *chemical pregnancy* (Table 2). *Intrapartum stillbirth* and *fetal anomaly/anomalies* were rated ‘unacceptable’ by 62.7% and 58.0% of respondents, whilst consensus on *fetal death* and *fetal loss* was even less clear; these were rated ‘unacceptable’ by 52.6% and 50.4% overall. In comparison, *stillbirth* used without ‘intrapartum’ was rated ‘unacceptable’ by only 13.6% of respondents in the same gestational brackets. The Wilcoxon signed‐rank test showed that the increased acceptability rating of *stillbirth* relative to *intrapartum stillbirth* was significant at these gestations (*z* = 7.879, *p* < 0.01).

The term *miscarriage* was rated ‘unacceptable’ by a marginal majority (61.2%) of respondents who had experienced loss between 18^+0^ and 23^+6^ weeks. However, at earlier gestations this terminology received mixed ratings, with 48.5% rating it ‘unacceptable’ (42.4% ‘acceptable’) at 14^+0^–17^+6^ weeks, and 82.6% rating it ‘acceptable’ for losses up to 5^+6^ weeks. Overall, 62.0% of respondents who had experienced loss before 24^+0^ weeks' gestation felt that *miscarriage* was ‘acceptable’ in healthcare settings. These mixed responses were reflected across all phrases including the word ‘miscarriage’ (*missed miscarriage, silent miscarriage, early miscarriage*, etc.) which respondents rated in the survey.

*Blighted ovum* and *empty sac* were rated ‘unacceptable’ by a marginal majority of respondents with experience of loss between 6^+0^ and 13^+6^ weeks' gestation, or when asked about diagnoses of anembryonic pregnancy, with means of 58.3% and 60.2% respectively. *Anembryonic pregnancy* received a mean unacceptability rating of 49.5% at these same gestations, showing significantly greater acceptability relative to *blighted ovum* (*z* = 4.048, *p* < 0.001) and *empty sac* (*z* = 3.493, *p* < 0.001).

For loss in early pregnancy, *chemical pregnancy* was rated ‘unacceptable’ by most respondents with experience of pregnancy loss before 5^+6^ weeks' gestation (59.9%). Phrases containing ‘viable’ were also generally rated unfavourably. Survey participants were asked about phrases containing *viable* both as a description of their experience (e.g., *non‐viable pregnancy*) and their baby (e.g., *non‐viable baby*). For gestational brackets up to 23^+6^ weeks, 57.7% of respondents rated this term ‘unacceptable’ as a description of their experience, while 72.1% of respondents rated this ‘unacceptable’ as a description of their baby.

### 3.3 Acceptable Terminology

As for unacceptability ratings, we categorised acceptability of terms according to degree of consensus among respondents (see Tables S1–S3). Words and phrases with a clear consensus on acceptability (rated ‘acceptable’ by 80.0%–100.0% of respondents) included *ectopic pregnancy, pregnancy loss* and *baby* (Table 3). *Ectopic pregnancy* was rated ‘acceptable’ by 91.2% of respondents with experience of extrauterine pregnancy. 80.0% also rated *pregnancy loss* as ‘acceptable’ in this context.

**TABLE 3:** Words and phrases rated ‘acceptable’ in the SuPPL dataset ordered by degree of consensus.

% rating	Acceptable words/phrases (*n* = total number of responses)			
80%–100% (clear consensus)	For the experience		For the baby	
	Ectopic pregnancy	91.2%, *n* = 57	Baby	93.8%, *n* = 485
	Stillbirth (after 24 weeks)	83.4%, *n* = 60	Their given name	84.7%, *n* = 470
	Pregnancy loss	81.6%, *n* = 272		
	Recurrent pregnancy loss (before 24 weeks)	81.3%, *n* = 75		
65%–80% (apparent consensus)	Born asleep/born sleeping (after 14 weeks)	79.8%, *n* = 142	Fetus (up to 13 weeks)	69.1%, *n* = 333
	Stillbirth (14–23 weeks)	76.3%, *n* = 82		
	Recurrent loss (before 24 weeks)	75.7%, *n* = 74		
50%–65% (marginal consensus)	Tubal pregnancy	62.3%, *n* = 53		
	Miscarriage (before 24 weeks)	62.0%, *n* = 211		
	Termination for Medical Reasons (for losses up to 30 weeks)	55.5%, *n* = 101		
	Extrauterine pregnancy	51.0%, *n* = 51		

Indeed, *pregnancy loss* was consistently rated by a clear majority of respondents as ‘acceptable’ at all gestations apart from after 40^+0^ weeks. This phrase was rated as ‘acceptable’ by 86.9% of respondents for use before 24^+0^ weeks' gestation. Only 62.0% felt that *miscarriage* was acceptable in the same contexts (*z* = 6.623, *p* < 0.001). *Recurrent pregnancy loss* and *recurrent loss* were also rated ‘acceptable’ by 81.3% and 75.7% respectively, of respondents with experience of multiple losses before 24^+0^ weeks.

Use of the term *stillbirth* to describe losses after 24^+0^ weeks' gestation, in line with UK guidelines, was also rated positively by a clear majority. 83.4% of respondents with experience of loss after 24^+0^ weeks' gestation rated *stillbirth* ‘acceptable’. Whilst not clinical nomenclature, *born asleep/born sleeping* met the 10.0% usage threshold for inclusion in the acceptability study, and were also consistently rated ‘acceptable’ by most participants who experienced loss after 14^+0^ weeks, with little variation according to the gestation at which loss occurred. Overall, 81.8% of responses rated these phrases ‘acceptable’ in healthcare settings across all stages of pregnancy after 14^+0^ weeks' gestation.

As noted above, a significant majority of respondents considered *baby* the most acceptable terminology to describe what they had lost, regardless of gestation at which a loss occurred. Across all gestational brackets, 93.8% of respondents rated *baby* ‘acceptable’, and only 7.1% rated it ‘unacceptable’. Lowest acceptability ratings for *baby* occurred in 30^+0^–39^+6^ week (89.6%) and post‐40^+0^ week (83.4%) gestational brackets, reflecting a preference for ‘their given name’; participants universally rated this option ‘acceptable’ from 24^+0^ weeks' gestation onwards. The comparative acceptability of ‘their given name’ by comparison with less acceptable *baby* is statistically significant (*z* = 3.453, *p* < 0.001).

Words and phrases rated ‘acceptable’ by 65.0%–79.0% of respondents (see Table 3) were all context specific. These included *stillbirth* for use between 14^+0^ and 23^+6^ weeks' gestation, *recurrent loss* for use before 24^+0^ weeks' gestation, and *fetus* up to 13^+9^ weeks' gestation. Many respondents who had experienced loss between 14^+0^ and 23^+6^ weeks considered *stillbirth* more acceptable than *miscarriage* in such contexts; 76.3% of respondents who experienced loss during these weeks rated *stillbirth* ‘acceptable’, by comparison with a 32.5% acceptability rating for *miscarriage* in the same gestational brackets. This difference is statistically significant (*z* = 5.508, *p* < 0.001).

For losses prior to 14^+0^ weeks' gestation, *fetus* was rated ‘acceptable’ by most respondents (69.1%). This is significantly lower than that for *baby*, which was considered ‘acceptable’ by 92.9% of respondents for losses up to 13^+9^ weeks' gestation. Comparison of these ratings showed *baby* had higher acceptability ratings than *fetus* in all gestational brackets up to 13^+6^ (*z* = 9.672, *p* < 0.001) and between 14^+0^ and 23^+6^ weeks' gestation (*z* = 7.201, *p* < 0.001).

Words and phrases deemed ‘acceptable’ by only a marginal majority (50.0%–64.0% of respondents) included *tubal pregnancy*, *extrauterine pregnancy* and *Termination for Medical Reasons* (Table 3). *Tubal pregnancy* was rated ‘acceptable’ by 62.3% of respondents with relevant experience, whilst *extrauterine pregnancy* was rated ‘acceptable’ by 51.0%. Respondents' ratings for *Termination for Medical Reasons (TFMR)* were mixed and varied by gestational bracket. For losses between 14^+0^ and 29^+6^ weeks' gestation, when most TFMR procedures take place [19], 55.0% of respondents rated the phrase ‘acceptable’, and only 34.0% rated it ‘unacceptable’. For losses from 30^+0^ weeks' gestation onwards, 66.7% considered it ‘unacceptable’. Overall, across all gestational brackets, this phrase was rated ‘acceptable’ by 48.9% of respondents and ‘unacceptable’ by 41.2% of respondents.

## 4 Discussion

### 4.1 Main Findings

This study demonstrates high levels of consensus among recent service users in the UK as to what pregnancy loss terminology is acceptable and what is unacceptable. By triangulating these quantitative findings with the wider EStELC project's qualitative insights, we can make evidence‐based recommendations for clinical terminology usage around pregnancy loss.

### 4.2 Strengths and Limitations

This pilot study provides the first empirical investigation of pregnancy loss terminology acceptability to service users in UK healthcare contexts. Survey materials were co‐produced by participants with lived experience of pregnancy loss and a diverse pool of healthcare practitioners involved in pregnancy loss care. This optimised the survey for coverage of diverse types of experience and comprehensiveness of terminology.

As a pilot study, the survey sample represents a small proportion of the wider population, and funding for further research to scale up these findings is being sought. Since the survey did not collect personal data, we cannot determine how well the sample reflects this population and some groups, including those underserved by research and at greater risk of perinatal loss [20, 21], may be underrepresented. Our volunteer sampling method also means that individuals with strong views on the topic may have been more motivated to take part. We therefore cannot exclude a selective response.

Smaller sample sizes for losses in later pregnancy, reflecting their relative infrequency compared to those in early pregnancy, make it more difficult to draw robust conclusions as to the acceptability of words to refer to losses after 24^+0^ weeks' gestation. Future research may need to use a different sampling model to avoid this pitfall. Survey responses, based on recollection of past experiences, may also be inaccurate. However, since responses concern the impact of terminology experienced, as well as the acceptability of terminology not necessarily encountered but which could be applied to losses of a similar type, participants' recall does not present a significant limitation.

### 4.3 Interpretation

Rejection of *spontaneous abortion* in the UK dates back at least 40 years [7, 22, 23], which explains why it was encountered by few respondents, with recall peaking at 14.2% for loss between 6^+0^ and 9^+0^ weeks' gestation. This may reflect its use, highlighted by EStELC participants [3], in health information on surgical management for first trimester loss, where overlap with termination of pregnancy services is not uncommon.

Phrases attributing ‘incompetence’ to the cervix have also long been acknowledged as offensive and potentially damaging [4, 5]. Whilst some endorse *cervical insufficiency* [6] instead, others have discerned little difference between labelling someone's cervix ‘incompetent’ and ‘insufficient’ [5, 10]. Indeed, study data showed little difference in the acceptability of *cervical insufficiency* and *incompetence*.

Likewise, *anembryonic pregnancy* and *blighted ovum* are highlighted in previous literature as divisive terminology [10, 24], whilst *empty sac* (along with *missed miscarriage*) was criticised strongly by EStELC participants. The finding that most respondents rated *blighted ovum* and *empty sac* as ‘unacceptable’ is thus consistent with previous considerations of these terms, though our dataset does not reflect the strength of negative feeling previous studies indicate. *Anembryonic pregnancy*, on which existing recommendations differ (cf. [10, 11]), is the most acceptable nomenclature for this experience.

Clinical jargon around pregnancy loss, including ‘fetal’, ‘intrapartum’, and ‘intrauterine’ consistently prompts negative reactions from a majority of respondents, even when combined with terminology such as ‘loss’ and ‘stillbirth’, which our findings show to be acceptable or neutral. Such patterns may reflect the EStELC findings that clinical language can be perceived by service users as ‘cold’ or ‘cruel’ [3, p. 81].

Ratings of unacceptability for *chemical pregnancy* align with the view that this phrase ‘implies that it was not a ‘real’ pregnancy, leading to anger and sometimes frustration on the part of parents’ [5, p. 1403] and reflects EStELC findings that this phrase was felt by those with lived experience of pregnancy loss to invalidate the loss [3, p. 44].

Respondents' ratings of *(non‐)viable* as an ‘unacceptable’ word for describing either the experience of loss or the baby itself align with concerns previously raised regarding this terminology [25, 26]. It has been noted that the ‘distinction between ‘pregnancy viability’ and ‘fetal viability’ indicates the need for care and clarity when using the term ‘viability’ in clinical practice and guidance’ [25, p. 725], since ‘telling someone being evaluated for early pregnancy that their ultrasonogram shows a viable pregnancy at 8 weeks of gestation can be confusing for someone who has heard the word viability applied in the context of [fetal viability]’ [25, p. 726]. The overlap in usage between these phrases, both often shortened to *viability*, may be related to the unacceptability of phrases including ‘viable*’* reported above.

Responses to *stillbirth* reflect findings of the EStELC project [3], which found low levels of dissatisfaction with this term. Respondents who had experienced second trimester loss considered *stillbirth* to be more acceptable than *miscarriage*, reflecting previous research findings on the difficulty the word *miscarriage* presents in contexts of late miscarriage [3, 26]. Only 22.4% of respondents who had experienced loss between 18^+0^ and 23^+6^ weeks' gestation rated *miscarriage* ‘acceptable’ for their experience. However, EStELC also found strong objections to *miscarriage* before 18^+0^ weeks' gestation, which are not reflected in this study's data. This difference may reflect disparities between EStELC and SuPPL cohorts, since EStELC's study design required a greater degree of commitment and input than SuPPL's and is thus likely to have attracted individuals who perceived terminology as a particularly damaging aspect of their pregnancy loss experience. Overall, *pregnancy loss* emerged as terminology most consistently felt to be ‘acceptable’, regardless of the gestation at which loss occurred.

*Termination for Medical Reasons* evoked mixed responses, with a marked rise in respondents rating *TFMR* as ‘unacceptable’ for losses after 30^+0^ weeks' gestation. This may reflect a limitation of the survey methodology, and further dedicated research is needed to explore terminology usage and preference around TFMR.

Finally, humanising labels such as *baby* and ‘their given name’ were consistently rated positively, while labels perceived as ‘dehumanising’ [3], such as *(pregnancy) tissue* and *products (of conception)*, were consistently rated negatively. Lower acceptability ratings for *baby* after 30^+0^ weeks' gestation is likely to reflect the preference for use of given names, whilst slightly lower acceptability ratings for *baby* before 12^+0^ weeks' gestation may reflect the preference of a minority of respondents to conceptualise the loss as something other than a baby (e.g., *embryo* or *fetus*).

By identifying both terminology rated ‘unacceptable’ and ‘acceptable’, this study was designed to identify alternatives for unacceptable nomenclature, using its evidence‐based consensus model (Table 4). For example, consensus was reached on *blighted ovum* as ‘unacceptable’ but *anembryonic pregnancy* as ‘acceptable’. Where no alternative to ‘unacceptable’ terms exists in the study's dataset, suggested alternatives are proposed in Table 4 on the basis of extrapolation from the findings of the EStELC and SuPPL projects.

**TABLE 4:** Recommended/suggested ‘acceptable’ alternatives to words and phrases found to be ‘unacceptable’.

Unacceptable language	Recommended alternative
Spontaneous abortion	Pregnancy loss/surgical management for pregnancy loss
Chemical pregnancy	Early pregnancy loss
Miscarriage	Pregnancy loss
Intrapartum fetal death	
Intrauterine death	
Intrapartum stillbirth	
Fetal death	
Fetal loss	
Blighted ovum	Anembryonic pregnancy
Empty sac	
Fetus	Baby

Unacceptable language	Suggested alternative
Incompetent cervix/cervical incompetence	Preterm cervical shortening
Cervical insufficiency	
Products of conception	Where necessary, for example when discussing removal of placental tissue or uterine lining following an incomplete loss, or in addition to the baby, consider using this language in an additive way, i.e., the baby and other pregnancy tissue
Contents of the womb/uterus	
Tissue	
Products	
Non‐viable (both baby and pregnancy)	
Pregnancy tissue	

## 5 Conclusion

This study has tested claims regarding harmful terminology for pregnancy loss and provided evidence of how this language is perceived by recent service users during experiences of pregnancy loss. On this basis, we have made recommendations for terminology use. These provide a starting point in transitioning to an empirical model of standardised terminology, which can minimise harm in mass communication contexts, where language is intended to be patient‐facing but cannot be individualised, as well as function optimally for clinical and research purposes. This transition should proceed in tandem with the implementation of a framework which accommodates individual language needs in interpersonal interactions.

## Author Contributions

B.M. obtained funding and conceived and designed the study. L.N. and B.M. collected the data and performed the analyses and interpretation of results. B.M. and L.N. wrote the manuscript which was revised collaboratively by all authors. The joint senior authors have made an equal contribution to this study and manuscript. All authors reviewed and approved the final manuscript.

## Ethics Statement

The SuPPL project received full ethics approval from the UCL Research Ethics Committee on 23 May 2024 (REC reference 26991/002).

## Conflicts of Interest

The authors declare no conflicts of interest.

## Supporting information


**Table S1:** Acceptability of most commonly used terms used to describe the experience of loss in relation to gestational bracket during which pregnancy loss occurred (*n* = total number of responses).
**Table S2:** Acceptability of most commonly used terms used to describe the baby in relation to gestational bracket during which pregnancy loss occurred (*n* = total number of responses).
**Table S3:** Acceptability of other commonly used terms used to describe specific experiences of loss.

## Data Availability

The data that support the findings of this study are available on request from the corresponding author. The data are not publicly available due to privacy or ethical restrictions.
