# Supplementary material for: Acceptable Nomenclature for Pregnancy Loss Care: A Cross‐Sectional Observational Survey
Source: BJOG. 2025 Oct 11;133(3):471–9. doi: 10.1111/1471-0528.70057 (PMC12770072; doi:10.1111/1471-0528.70057)
Supplement: Supplementary file 1 — Table S1: Acceptability of most commonly used terms used to describe the experience of loss in relation to gestational bracket during which pregnancy loss occurred (n = total number of responses). Table S2: Acceptability of most commonly used terms used to describe the baby in relation to gestational bracket during which pregnancy loss occurred (n = total number of responses). Table S3: Acceptability of other commonly used terms used to describe specific experiences of loss. [file BJO-133-471-s001.docx]

**Supplementary material (to be published online)**

Tables S1 and S2 list the words and phrases most commonly recalled by SuPPL participants (recalled by at least 10% of participants asked in response to any one question) as being used to describe experiences of pregnancy loss (Table S1) and the baby itself (Table S2) in UK healthcare settings.

***Table S1: Acceptability of most commonly used terms used to describe the experience of loss in relation to gestational bracket during which pregnancy loss occurred (n= total number of responses).***

| **Gestational period at which pregnancy loss occurred (weeks)** | **1-5** | **6-9** | **9-13** | **14-17** | **18-23** | **24-29** | **30-39** | **40+** |
| --- | --- | --- | --- | --- | --- | --- | --- | --- |
| **Miscarriage**  % ‘Unacceptable’ (1-3)  % ‘Acceptable’ (5-7) | (*n=127*)  10.2  82.6 |  |  | (*n= 33*)  48.5  42.4 | (*n=49*)  61.2  22.4 | (*n=18*)  55.6  38.4 | (*n=21*)  81.0  19 |  |
| **Pregnancy loss**  % ‘Unacceptable’ (1-3)  % ‘Acceptable’ (5-7) | (*n=127*)  2.4  92.9 |  |  | *(n=35)*  11.5  85.7 | *(n=51)*  13.7  72.6 | *(n=18)*  16.7  77.7 | *(n=26)*  23  69.1 | *(n=12)*  66.7  25 |
| **Stillbirth**  % ‘Unacceptable’ (1-3)  % ‘Acceptable’ (5-7) |  |  | *(n=64)*  42.2  45.4 | *(n=32)*  12.5  71.9 | *(n=48)*  18.8  79.2 | *(n=19)*  10.6  89.5 | *(n=29)*  10.3  79.3 | *(n=12)*  8.3  83.4 |
| **Born asleep/born sleeping**  % ‘Unacceptable’ (1-3)  % ‘Acceptable’ (5-7) |  |  |  | *(n=34)*  17.7  79.3 | *(n=49)*  12.3  81.7 | *(n=19)*  5.3  94.7 | *(n=28)*  14.3  82.9 | *(n=12)*  33.3  66.7 |
| **Non-viable pregnancy**  % ‘Unacceptable’ (1-3)  % ‘Acceptable’ (5-7) | (*n=129*)  54.2  27.2 | (*n=126*)  52.4  33.4 | (*n=66*)  51.6  34.9 | (*n=32*)  75  25.7 | (*n=49*)  77.5  16.4 |  |  |  |
| **Termination For medical reasons**  % ‘Unacceptable’ (1-3)  % ‘Acceptable’ (5-7) |  |  |  | (*n=33*)  27.3  57.7 | (*n=50*)  36  52 | (*n=16*)  43.8  56.3 | (*n=20*)  55  35 | (*n=10*)  90  10 |
| **Intrauterine death**  % ‘Unacceptable’ (1-3)  % ‘Acceptable’ (5-7) |  |  |  | (*n=29)*  62  20.6 | (*n=47)*  72.3  14.9 | (*n=16)*  50  37.5 | (*n=23)*  73.9  8.6 | (*n=12)*  58.4  16.6 |
| **Intrapartum fetal death**  % ‘Unacceptable’ (1-3)  % ‘Acceptable’ (5-7) |  |  |  | (*n=30)*  73.3  20 | (*n=47)*  76.6  12.7 | (*n=16)*  50.1  25 | (*n=22)*  77.2  4.5 | (*n=12)*  50.1  33.4 |
| **Intrapartum stillbirth**  % ‘Unacceptable’ (1-3)  % ‘Acceptable’ (5-7) |  |  |  | (*n=29)*  62  17.2 | (*n=47)*  72.3  17.1 | (*n=16)*  50.1  31.3 | (*n=22)*  59.1  18.1 | (*n=12)*  49.9  33.4 |
| **Fetal anomaly/anomalies**  % ‘Unacceptable’ (1-3)  % ‘Acceptable’ (5-7) |  |  |  | (*n=34)*  47  41.2 | (*n=48)*  54.2  31.3 | (*n=17)*  52.9  35.3 | (*n=21)*  71.4  14.3 | (*n=11)*  90.9  9.1 |
| **Fetal death**  % ‘Unacceptable’ (1-3)  % ‘Acceptable’ (5-7) |  |  |  | (*n=33)*  54.6  42.4 | (*n=47)*  61.6  29.7 | (*n=18)*  16.8  50.1 | (*n=25)*  60  24 | (*n=12)*  49.9  25 |
| **Fetal loss**  % ‘Unacceptable’ (1-3)  % ‘Acceptable’ (5-7) |  |  |  | (*n=34)*  44.1  47.1 | (*n=47)*  55.3  34.1 | (*n=17)*  23.5  52.9 | (*n=25)*  56  28 | (*n=12)*  75  8.3 |
| **Spontaneous abortion**  % ‘Unacceptable’ (1-3)  % ‘Acceptable’ (5-7) |  | (*n=125*)  90.4  6.4 | (*n=67*)  83.6  10.5 | (*n=30*)  90  6.6 | (*n=46*)  91.3  6.5 |  |  |  |
| **Incompetent cervix**  % ‘Unacceptable’ (1-3)  % ‘Acceptable’ (5-7) |  |  |  | (*n=30*)  79.9  13.3 | (*n=47*)  80.8  12.8 | (*n=16*)  93.8  6.3 | (*n=21*)  85.8  0 |  |
| **Cervical incompetence**  % ‘Unacceptable’ (1-3)  % ‘Acceptable’ (5-7) |  |  |  | (*n=32*)  84.5  9.4 | (*n=48*)  79.2  12.5 | (*n=16*)  87.5  6.3 | (*n=22*)  86.3  0 |  |
| **Anembryonic pregnancy**  % ‘Unacceptable’ (1-3)  % ‘Acceptable’ (5-7) |  | (*n=115)*  52.2  36.5 | (*n=65)*  44.7  38.4 |  |  |  |  |  |
| **Blighted ovum**  % ‘Unacceptable’ (1-3)  % ‘Acceptable’ (5-7) |  | (*n=119)*  61.3  26.9 | (*n=66)*  53  27.3 |  |  |  |  |  |
| **Empty sac**  % ‘Unacceptable’ (1-3)  % ‘Acceptable’ (5-7) |  | (*n=124)*  62.1  29 | (*n=66)*  56.1  30.3 |  |  |  |  |  |
| **No fetal pole**  % ‘Unacceptable’ (1-3)  % ‘Acceptable’ (5-7) |  | (*n=118)*  62.7  25.4 | *(n=65)*  60.1  23.1 |  |  |  |  |  |
| **Chemical pregnancy**  % ‘Unacceptable’ (1-3)  % ‘Acceptable’ (5-7) | (*n=125)*  55.2  28.8 |  |  |  |  |  |  |  |

***Table S2: Acceptability of most commonly used terms used to describe the baby in relation to gestational bracket during which pregnancy loss occurred (n= total number of responses).***

| **Gestational period at which pregnancy loss occurred (weeks)** | **1-5** | **6-9** | **9-13** | **14-17** | **18-23** | **24-29** | **30-39** | **40+** |
| --- | --- | --- | --- | --- | --- | --- | --- | --- |
| **Baby**  % ‘Unacceptable’ (1-3)  % ‘Acceptable’ (5-7) | (*n=130)*  5.3  93.1 | (*n=136)*  3.7  92.6 | (*n=72)*  2.8  93.1 | (*n=35)*  0  100 | (*n=52)*  1.9  98 | (*n=19)*  0  100 | (*n=29)*  6.8  89.6 | (*n=12)*  0  83.4 |
| **Their given name**  % ‘Unacceptable’ (1-3)  % ‘Acceptable’ (5-7) | (*n=126)*  11.9  79.4 | (*n=126)*  15.1  77.9 | (*n=69)*  17.3  79.6 | (*n=34)*  0  100 | (*n=52)*  3.8  96.1 | (*n=19)*  0  100 | (*n=28)*  0  100 | (*n=12)*  0  100 |
| **Fetus**  % ‘Unacceptable’ (1-3)  % ‘Acceptable’ (5-7) | (*n=128)*  14.8  71.1 | (*n=137)*  17.6  67.9 | (*n=71)*  18.3  67.6 | (*n=34)*  52.9  32.4 | (*n=50)*  66  28 | (*n=18)*  94.4  5.6 | (*n=23)*  91.3  4.3 | (*n=12)*  100  0 |
| **Contents of the uterus/ womb**  % ‘Unacceptable’ (1-3)  % ‘Acceptable’ (5-7) | (*n=127)*  85.8  10.1 | (*n=132)*  78.8  14.3 | (*n=71)*  69.1  18.3 | (*n=35)*  94.3  5.7 | (*n=50)*  94  6 |  |  |  |
| **Pregnancy tissue**  % ‘Unacceptable’ (1-3)  % ‘Acceptable’ (5-7) | (*n=128)*  73.5  20.3 | (*n=135)*  69.6  20.6 | (*n=72)*  66.7  23.6 | (*n=35)*  94.3  0 | (*n=50)*  88  6 |  |  |  |
| **Products of conception**  % ‘Unacceptable’ (1-3)  % ‘Acceptable’ (5-7) | (*n=126)*  88.9  7.2 | (*n=136)*  84.6  10.3 | (*n=72)*  79.1  9.8 | (*n=35)*  97.2  0 | (*n=50)*  94  6 |  |  |  |
| **Products**  % ‘Unacceptable’ (1-3)  % ‘Acceptable’ (5-7) | (*n=127)*  98.4  0.8 | (*n=132)*  93.9  6.1 | (*n=69)*  91.2  5.7 | (*n=34)*  100  0 | (*n=50)*  94  6 |  |  |  |
| **Tissue**  % ‘Unacceptable’ (1-3)  % ‘Acceptable’ (5-7) | (*n=128)*  88.4  3.9 | (*n=133)*  86.5  7.6 | (*n=71)*  78.9  14 | (*n=34)*  100  0 | (*n=50)*  96  2 |  |  |  |
| **Non-viable**  % ‘Unacceptable’ (1-3)  % ‘Acceptable’ (5-7) | (*n=126)*  69  19.9 | (*n=134)*  68.7  23.2 | (*n=68)*  67.6  25 | (*n=34)*  82.3  8.8 | (*n=50)*  88  8 |  |  |  |
| **A stillborn**  % ‘Unacceptable’ (1-3)  % ‘Acceptable’ (5-7) |  |  |  |  | (*n=50)*  38  52 | (*n=18)*  38.9  55.6 | (*n=28)*  49.9  28.6 | (*n=12)*  75  25 |
| **Cells**  % ‘Unacceptable’ (1-3)  % ‘Acceptable’ (5-7) | (*n=128)*  88.3  4.7 | (*n=133)*  80.5  12.1 | (*n=70)*  84.3  10 |  |  |  |  |  |
| **Mass**  % ‘Unacceptable’ (1-3)  % ‘Acceptable’ (5-7) | (*n=127)*  93.7  4 | (*n=131)*  93.1  4.6 | (*n=70)*  90  7.1 |  |  |  |  |  |

***Table S3: Acceptability of other commonly used terms used to describe specific experiences of loss***

| **Specific type of pregnancy loss experience** | **Ultrasound showed a pregnancy sac but no baby was visible** | **Baby implanted outside of uterus** | **2 or more losses before 24 weeks** |
| --- | --- | --- | --- |
| **Anembryonic pregnancy**  % ‘Unacceptable’ (1-3)  % ‘Acceptable’ (5-7) | (*n=82)*  47.6  43.9 |  |  |
| **Blighted ovum**  % ‘Unacceptable’ (1-3)  % ‘Acceptable’ (5-7) | (*n=84)*  61.9  43.9 |  |  |
| **Empty sac**  % ‘Unacceptable’ (1-3)  % ‘Acceptable’ (5-7) | (*n=86)*  57  28 |  |  |
| **Ectopic pregnancy**  % ‘Unacceptable’ (1-3)  % ‘Acceptable’ (5-7) |  | (*n=57)*  1.8  91.2 |  |
| **Ruptured ectopic**  % ‘Unacceptable’ (1-3)  % ‘Acceptable’ (5-7) |  | *(n=54)*  20.4  74 |  |
| **Extrauterine pregnancy**  % ‘Unacceptable’ (1-3)  % ‘Acceptable’ (5-7) |  | *(n=51)*  39.2  51 |  |
| **Tubal pregnancy**  % ‘Unacceptable’ (1-3)  % ‘Acceptable’ (5-7) |  | *(n=53)*  24.6  62.3 |  |
| **Fertility problems/issues**  % ‘Unacceptable’ (1-3)  % ‘Acceptable’ (5-7) |  |  | *(n= 72)*  38.9  47.2 |
| **Recurrent loss**  % ‘Unacceptable’ (1-3)  % ‘Acceptable’ (5-7) |  |  | *(n= 74)*  16.3  75.7 |
| **Recurrent pregnancy loss**  % ‘Unacceptable’ (1-3)  % ‘Acceptable’ (5-7) |  |  | *(n= 75)*  11.9  81.3 |
| **Recurrent miscarriage**  % ‘Unacceptable’ (1-3)  % ‘Acceptable’ (5-7) |  |  | *(n= 74)*  23.5  74.3 |
